# Nanometer collimation enhancement of ion beams using channeling effects in track-etched mica capillaries

**DOI:** 10.1038/s41598-017-17005-w

**Published:** 2017-12-06

**Authors:** Clemens Scheuner, Steffen Jankuhn, Jürgen Vogt, Sébastien Pezzagna, Christina Trautmann, Jan Meijer

**Affiliations:** 1 Universität Leipzig, Faculty of Physics and Earth Sciences, Linnéstraße 5, 04103 Leipzig, Germany; 2 Joint Single Ion Implantation Lab, 04318 Leipzig, Germany; 3 GSI Helmholtzzentrum für Schwerionenforschung, 64291 Darmstadt, Germany; 4 Technische Universität Darmstadt, 64287 Darmstadt, Germany

## Abstract

Long channels with diameter of few tens of nanometer are produced by chemical track etching of swift heavy ion irradiated muscovite sheets. Such small apertures are most suitable e.g. as beam defining apertures for focusing systems in ion beam facilities enabling beam diameters down to a few nanometers. One of the most important parameters to consider is the interaction of the ion beam with the walls of the aperture. We report angle-resolved transmission and energy-loss measurements of MeV ion beams through ion-track-etched capillaries with very high aspect ratio of about 60. For all ion energies, the angle-resolved transmission curves measured through the channels show a significant enhancement with respect to the expected pure geometrical considerations. This broadening of the acceptance angle increases further when the kinetic energy is reduced. This effect is ascribed to low-angle scattering of the ions at the surface of the muscovite capillary walls. These results are well described by simulations applying a similar approach as used for ion beam channeling in crystals.

## Introduction

Small and robust apertures are important for a large number of applications especially in ion beam technology. The collimation of particles like ions with energies in the keV to MeV range requires a mask of several μm thickness. Plasma etching or milling with a focused ion beam (FIB) are in principle suitable methods to fabricate tiny holes^1^, but the aspect ratio achieved by these techniques is limited, and not suitable for thick mask material. One technique to overcome this problem is the ion track nanotechnology using swift heavy ions of kinetic energy in the MeV to GeV range. In many materials, mainly insulators, such ion projectiles produce along their trajectories a few nanometer wide track consisting of damaged, often amorphous material^2^. Employing a suitable chemical etching, the tracks can be converted into open channels. The diameter depends on the applied etching parameters and etching time. Given the high kinetic energy, this procedure can be used for thick films of several tens of μm^3^. Apertures produced by ion track etching have already been successfully used as irradiation mask for example to create a pair of close-by Nitrogen-Vacancy (NV) centres in diamond having been entangled at room temperature^4,5^. Track-etched polymer membranes were also successfully applied as templates to grow tailored nanowires^6^.

A suitable and promising material to create small pores by means of ion-track etching is muscovite mica because of its high chemical stability. Muscovite mica has a complex crystal structure with chemical formula ${\text{KAl}}_{2}[{\text{(OH)}}_{2}|{\text{AlSi}}_{3}{\text{O}}_{10}]$. Track-etched capillaries have a rhombus-shaped cross-section with very sharp edges and smooth side walls^3,7^. The latter is a very important requirement for apertures used for ion beam collimation of low MeV ions. The smoothness defines the so called transparent zone, which is a section near the edges where the ions pass through the aperture being deflected but not being stopped. If the ion beam has a certain divergence and the thickness of the sheet is high, the smoothness of the edge is even more important. Additionally, the mask material must be radiation hard and should not be degenerated when exposed to the ion beam. For highly charged ions it has been found that charging up of the channel walls leads to a focusing effect^8^. Furthermore, charging of pore walls was observed for ions below 100 keV^9^.

Compared to geometric considerations, an unexpected surplus of singly and doubly charged ions passing through the channels within small entrance angles was observed. To demonstrate our assumption of small angle scattering inside the capillaries, we performed experiments at different kinetic energies and tilt angles.

## Results

**Figure 1:**
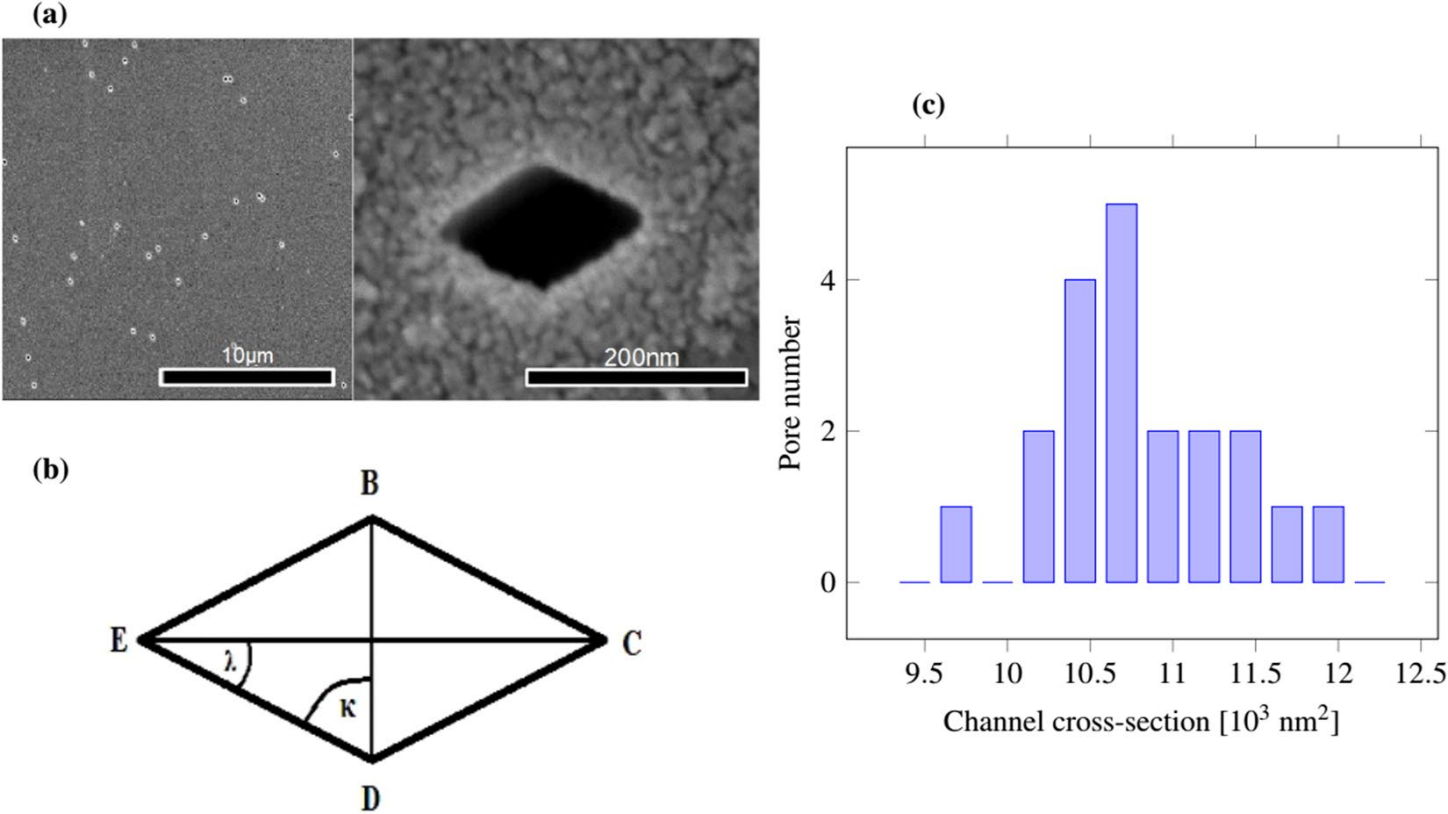
(**a**) Scanning electron images of track-etched channels at low and high magnification. (**b**) Scheme of channel cross-section with average shape size $b=\overline{{\rm{BD}}}=112\pm 11$ nm, $c=\overline{{\rm{EC}}}=203\pm 16$ nm and $a=\overline{{\rm{E}}{\rm{B}}}=\overline{{\rm{B}}{\rm{C}}}=\overline{{\rm{C}}{\rm{D}}}=\overline{{\rm{D}}{\rm{E}}}=115\pm 22$ nm. The angles are $2\kappa =117\pm {8}^{\circ }$ and $2\lambda =63\pm {4}^{\circ }$. (**c**) Channel cross-section *A* after track etching deduced from SEM images of 20 channels.

From scanning electron microscopy (SEM) micrographs (Fig. 1) we can measure the number of pores per area and the mean cross-section area of the pores. We obtain a porosity of $\mathrm{(3.4}\pm \mathrm{0.2)}\times {10}^{-4}$. This open area can be compared to the maximum membrane transmission of 2 MeV N^2+^ ions and direct irradiation of the detector. The ratio of the two measurements gives $\frac{{Y}_{{\rm{sheet}}}{\mathrm{(0}}^{o})}{{Y}_{{\rm{direct}}}}=1.5\times {10}^{-4}$. From this two values a transmission yield (at 0°) of 44 ± 8% is deduced.

The difference between the measured yield and the porosity may have different reasons: (i) The edges of the pores are possibly not completely smooth, or slightly misaligned, (ii) there might be some residual gas or water within the pores, and (iii) the ion current measurement may be underestimated due to a detector efficiency that decreases for high ion currents. Nevertheless the transmission measurements are in the same order of magnitude as the porosities, and seem to be reasonable.

**Figure 2:**
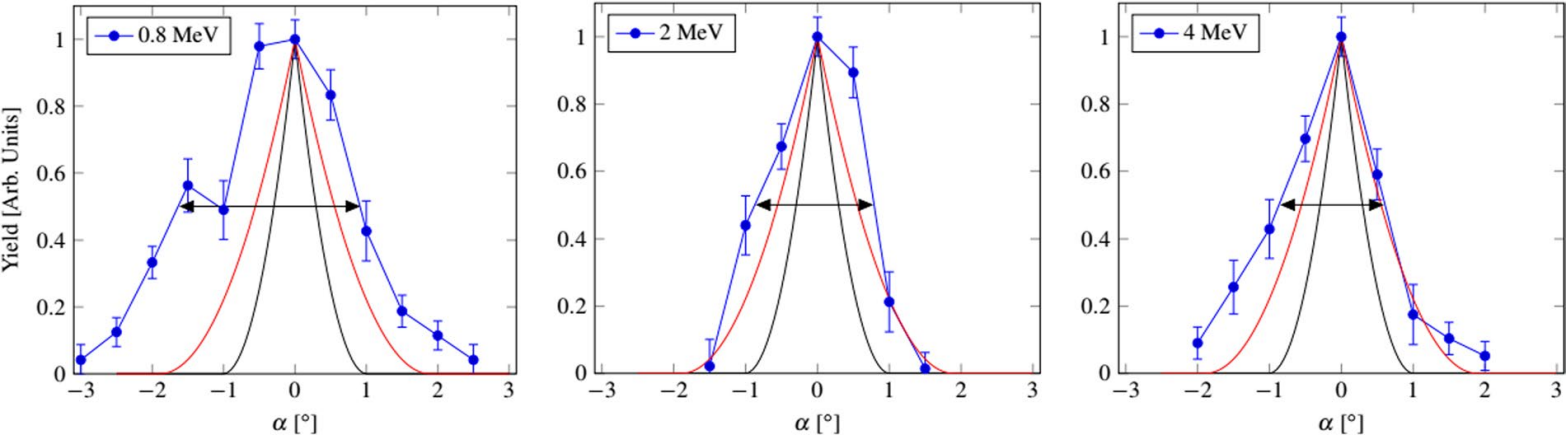
Measured transmission yield as a function of the tilt angle for three different ion energies. The transmission expected from geometric considerations only is also plotted for the short (—) and long () axis of the rhombus-shaped pores. The black arrow shows the full width at half maximum *β*.

To test transmission under different angles of beam incidence, the tilt angle *α* was varied in small steps and the number of ions transmitted per step was integrated. Figure 2 shows the normalized transmission yield as a function of the tilt angle *α*. This measurement was done for three ion energies. For further analysis we considered *β* as the angle of full width at half maximum (FWHM) of the transmission. The resulting values are $\beta ={\mathrm{(2.6}\pm \mathrm{0.2)}}^{\circ }$ for 0.8 MeV, $\beta ={\mathrm{(1.66}\pm \mathrm{0.14)}}^{\circ }$ for 2 MeV and $\beta ={\mathrm{(1.50}\pm \mathrm{0.12)}}^{\circ }$ for 4 MV. This is displayed in Fig. 2. The 8% error includes the uncertainties in the angle of the goniometer and the number of recorded events per measurement and thus the statistical error.

To get a qualitative understanding of the transmission behavior of the ions through the sheet, the geometric effect of rotating the capillaries with respect to the ion beam was taken into account. The capillaries have a rhombus-shaped cross section of average width of $b=\mathrm{(112}\pm \mathrm{11)}$ nm and $c=\mathrm{(203}\pm \mathrm{16)}$ nm and a length equal to the sheet thickness of $t=\mathrm{(7}\pm \mathrm{0.5)}$ μm. Tilting the sheet results in a reduction of the open effective cross section. The maximum opening is at 0° where the capillaries are perfectly aligned with respect to the incident beam. If the sheet gets tilted, the open cross section reduces as a function of the tilting angle *α*

$${Y}_{{\rm{g}}{\rm{e}}{\rm{o}}{\rm{m}}}(\alpha )={(\frac{h-\tan \alpha \cdot t}{2\cos \omega })}^{2}\sin 2\omega $$

where *t* is the thickness of the sheet and *h* is the diagonal of a pore, this can be either *b* or *c* depending on the pore orientation. *ω* is the corresponding angle at the diagonal, this is *λ* for diagonal *c* and *κ* for diagonal *b*. The calculated cross sections as a function of *α* are included in Fig. 2.

When comparing the calculated and the experimental yield data, it is obvious that the measured curves are systematically broader, even if the maximum of the pore size distribution is taken into account. This effect increases with lower energies. Geometric effects alone can obviously not explain this broadening suggesting another effect that enhances the transmission of the ions through the capillaries. We propose this to be crystal channeling. This is a well known effect of ion implantation processes in crystals occurring if the ions move under a small angle with respect to certain specific crystal directions. The ions get deflected along the plane leading to enhanced penetration depths. In this case the potential of the crystal atoms can be assumed to be continuous because at high ion velocities, the potential of the single atoms averages to a smooth potential^10^. Channeling is expected to be possible for ions traveling close to the wall of capillaries, because the ion trajectories form a small angle with the smooth surface walls. After etching, the inner walls are expected to consist of mono crystalline oxygen-terminated planes because they have the slowest etching rate. This planes form a rhombus with angles of 60° and 120° in the mica unit cell^7^, similar to the rhombus-shape of the pore cross-section observed in the SEM micrographs shown in Fig. 1a.

To simulate channeling, two different scattering possibilities, axial and planar, can be considered. In the axial case, we assume a straight row of atoms on which the ions are scattered. This leads to a mixture of scattering events at the different atomic constituents of mica, with an atomic number of 9.43 as stoichiometric average. Under planar scattering the ion gets deflected under a small angle on the oxygen planes dominating the wall of the pore. So the atomic number of the target atoms *Z*_2_ is set to 8 for oxygen. If the ions are assumed to penetrate the first layer of atoms and get scattered at an arbitrary position in the crystal, again the stoichiometric average of 9.43 has to be considered. However, this would lead just to small deviations that have no influence on the interpretation of the data.

To describe the deflection angle ${{\rm{\Psi }}}_{p}$ between target atoms and projectile ions we used the formula for the characteristic angle for planar channeling^10^:

$${{\rm{\Psi }}}_{{\rm{p}}}=\sqrt{\frac{{Z}_{1}{Z}_{2}n{e}^{2}a}{2{\varepsilon }_{0}E}}$$

*Z*_1_ denotes the atomic number of the projectile ion which is 7 for nitrogen, *Z*_2_ is in the planar case 8 for oxygen and $n=\mathrm{1/(7.28)}$ Å^−2^ is the atomic areal density in the plane according to the data given in^11^. Furthermore *α* is the Thomas-Fermi screening radius:

$$a=0.8853\cdot {a}_{0}{(\sqrt{{Z}_{1}}+\sqrt{{Z}_{2}})}^{-\frac{2}{3}}$$

with the Bohr radius ${a}_{0}=0.528$ Å.

For axial channeling, the deflection angle ${{\rm{\Psi }}}_{{\rm{a}}}$ is given by^10^:

$${{\rm{\Psi }}}_{{\rm{a}}}=\sqrt{\frac{{Z}_{1}{Z}_{2}{e}^{2}}{2\pi d{\varepsilon }_{0}E}}$$

where *d* is the distance between the ions in a row of atoms. Due to the complicated crystal structure of muscovite, there are many different possibilities for scattering atoms in a row. Therefore the calculation was simplified by taking the muscovite density of 2.83 $\frac{{\rm{g}}}{{{\rm{cm}}}^{3}}$ and averaging the atomic distance to *d* = 2.23 Å. The mixing of the characteristic angles for the elements of the mica crystal is assumed to happen in a way that the characteristic angle is calculated for every element and then averaged over all characteristic angles in the following way:

$${{\rm{\Psi }}}_{a,{\rm{a}}{\rm{v}}{\rm{e}}{\rm{r}}{\rm{a}}{\rm{g}}{\rm{e}}}=\sum _{i=1}^{n}\frac{{{\rm{\Psi }}}_{a,i}\cdot {k}_{i}}{n}$$

where *i* is the index for each element and *k*_*i*_ is the stoichiometry of mica.

The distance between ion and target atom is the main parameter for the deflection behavior. This distance varies due to thermal motion of the target atoms. For stronger thermal amplitudes, larger distances between ions and target surface are needed for stable channeling. The thermal movement can be estimated as follows^10^:

$$\rho =12.1\sqrt{\frac{\frac{{\rm{\Phi }}}{x}+\frac{1}{4}}{M\cdot {\rm{\Theta }}}}{\AA}$$

with Θ being the Debye temperature, $x=\frac{{\rm{\Theta }}}{T}$ and ${\rm{\Phi }}(x)=\frac{1}{x}{\int }_{0}^{x}{\rm{d}}\xi \frac{\xi }{{e}^{\xi }-1}$ is the Debye-function. *M* denotes the atomic mass in atomic units and the target temperature *T* is in our measurements room temperature. According to^12^, the Debye temperature for oxides and silicates is in between 800 and 1200 K. Under this condition, the thermal movement has an amplitude of $\rho =(0.045\mathrm{\mbox{--}}0.067)$ Å. We are now able to calculate the probability distribution for either planar (*Y*_*p*_) or axial (*Y*_*a*_) channeling^10^:

$${Y}_{{\rm{c}}{\rm{h}}{\rm{a}}{\rm{n}}}=1-\exp [(-{C}^{2}{a}^{2}/{u}^{2})/(\exp (2{\alpha }^{2}/{{\rm{\Psi }}}_{{\rm{a}}/{\rm{p}}}^{2})-1)]$$

where $C=\sqrt{3}$ is an empirical constant and $u=\sqrt{2}\rho$ is the root mean square value of the thermal vibration amplitude *ρ*.

To compare the calculated and experimental values, we need to consider the geometric effect and the channeling distribution. Under perfect alignment of the capillary axis along the ion beam direction, the ions will not interact with the pore wall. When tilting, the geometric open cross-section gets smaller and some ions collide with the walls. For these collisions there is a certain probability for channeling and thus back scattering of ions from the pore wall, depending on the tilt angle. This leads to the ion transmission as a function of the tilt angle:

$$Y(\alpha )=({Y}_{{\rm{g}}{\rm{e}}{\rm{o}}{\rm{m}}}(\alpha )+[1-{Y}_{{\rm{g}}{\rm{e}}{\rm{o}}{\rm{m}}}(\alpha )]{Y}_{{\rm{c}}{\rm{h}}{\rm{a}}{\rm{n}}}(\alpha ))\cos \,\alpha $$

**Figure 3:**
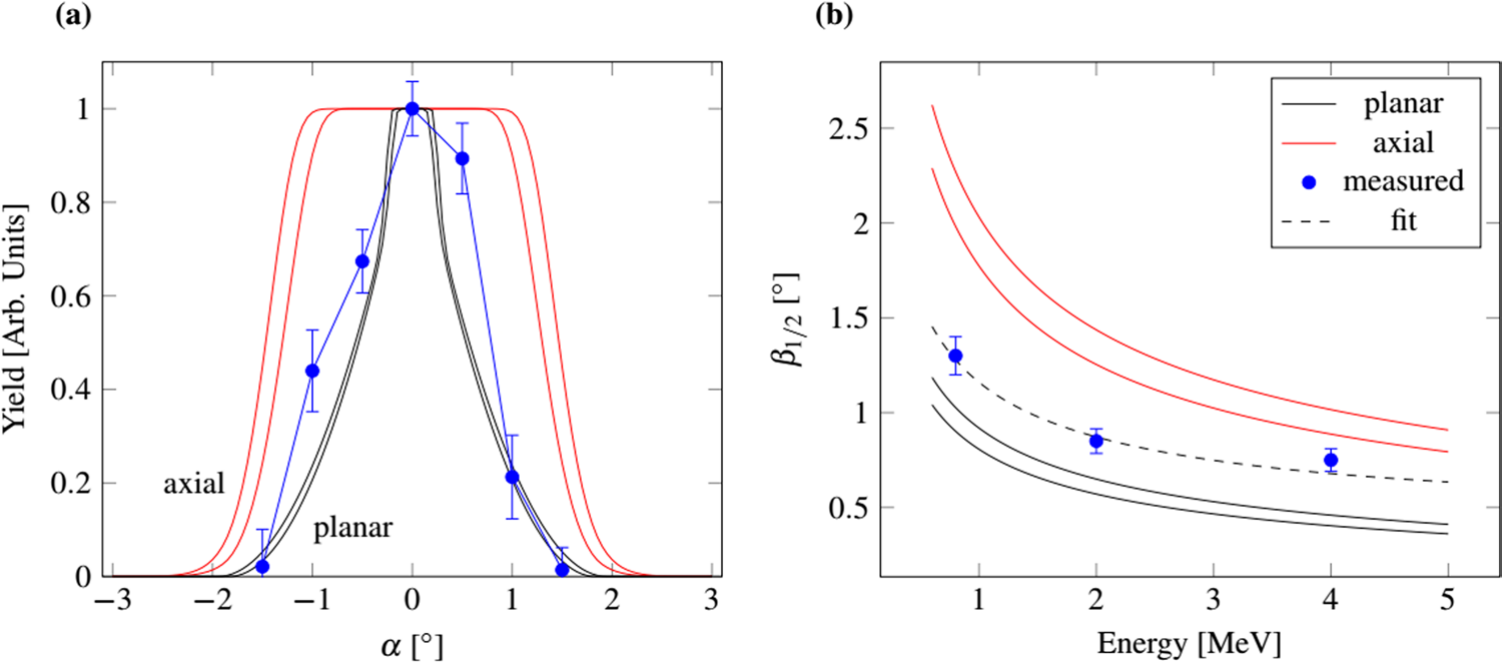
**(a)** Transmission yield as a function of tilt angle *α* for 2 MeV N^2+^ () projectiles. The solid lines are the calculated minimum and maximum values for axial () and planar (*—*) channeling, respectively. **(b)** FWHM angle *β* as a function of projectile energy for the measured data and calculated (min, max) distributions for planar and axial channeling. The error region is between the two lines. The dashed line is a fit to the experimental data.

The corresponding distributions together with experimental data are shown in Fig. 3.

The FWHM angle *β* of the yield vs. tilt angle is deduced by solving $Y(\psi ,E)=0.5$ (Fig. 3). The measured data is in between the calculated curves for axial and planar channeling. This strengthens our assumption that channeling is the cause of the broadened transmission angles that we observe in Fig. 2. The experimental data are slightly closer to the planar calculation. Given the complicated crystal structure of muscovite, it seems reasonable that, ion channeling along planes is more probable than parallel to a crystal row. The dashed gray line in Fig. 3b corresponds to a fit to the experimental data giving evidence that the shape of the curve is the same as for planar channeling.

Planar channeling seems to describe the trajectory of the ions rather well for MeV energies and low charged ions. Furthermore, the energy loss of the ions while traveling trough the pores was recorded in dependence of the tilt angle. For larger tilt angles *α*, the energy loss increases and the transmission becomes very small. Experimentally, this situation becomes difficult due to the small number of detected ions and the corresponding large statistical uncertainties. To estimate the energy loss, the ion trajectories were simulated as a straight path in the pores until the ions hit the wall. For the incidence angle *α*, the number of scattering events *S* can be calculated by: $S=t\cdot \,\tan \,\alpha /d$ where *t* is the thickness of the muscovite sheet and *d* is an effective radius equal to the radius of a circle with the same area as the pore, which is about (60 ± 2) nm. To calculate the energy loss for each scattering event, the kinematic formula from Rutherford-back-scattering experiments was used^13^:

$${E}_{{\rm{l}}{\rm{o}}{\rm{s}}{\rm{s}}}={E}_{{\rm{i}}{\rm{n}}}(1-{[\frac{\sqrt{1-{(\frac{{M}_{1}\cdot \sin \alpha }{{M}_{2}})}^{2}}+\frac{{M}_{1}\cdot \cos \alpha }{{M}_{2}}}{1+\frac{{M}_{1}}{{M}_{2}}}]}^{2})$$

where *E*_in_ is the incident ion energy and *M*_1_ and *M*_2_ denote the mass of projectile and target atoms, respectively. Calculation yields just a very small energy loss in the range of 1.5% of the incident ion energy. This energy loss is smaller than the respective experimental error and this further strengthens the assumption of crystal channeling. For angles so large that the pore is geometrically closed, the ions need to penetrate the material for a certain distance if no guiding is assumed. This would lead to a significant energy loss.

## Discussion

Transmission experiments of MeV light ions through high-aspect ratio nanochannels in mica show unexpected transmission yields when tilting, which can not be explained by geometric means. We ascribe this effect to channeling of ions within the surface walls of the capillaries. We provide evidence that charging effects are not influencing the pathway of single and double charged ions in the MeV range. The potential along the capillary is constant and the electric field inside the pore is negligible. Additionally, we coated the surface of the mica sheet with a metal layer. Knowing from Wang *et al*.^9^ and Zhang *et al*.^8^ that charging of nanopores influences the transmission of low-energy ions and could also lead to a guiding effect, it is expected that electric effects get more pronounced for ions with greater charges or lower energies. For ions with several 100 keV charging up becomes less important. The experimental data are in good agreement with calculations for axial and planar channeling. Track-etched channels in muscovite sheets are shown to be radiation hard and ideal nanoapertures for ion beams in the MeV regime. This opens new fields of applications such as mapping of radiation hardness by means of ion beam induced current (IBIC) measurements with high lateral resolution or targeted irradiation of single bio cells. Moreover, nanochannel collimation is a suitable, low cost alternative to microprobe systems for low current applications or single ion implantation.

## Methods

Muscovite sheets (provided by Jahre GmbH) of 5–10 μm thickness were irradiated at the linear accelerator UNILAC of the GSI Helmholzzentrum in Darmstadt^3^. The irradiations where performed with Samarium ions under normal beam incidence and a kinetic energy of 1.6 GeV corresponding to a range of 90 μm in mica^14^. Since each individual ion creates a track, the number of tracks is controlled by the applied ion fluence. For this experiment we used a fluence of $\mathrm{(3}\pm \mathrm{0.15)}\times {10}^{6}$ ions per cm^2^. Room temperature etching of irradiated mica samples in 10% hydrofluoric acid for one hour leads to highly parallel channels with rhombic cross-section. Figure 1 shows SEM images of the track-etched mica at low and high magnification together with the scheme of a single pore cross-section as well as the shape distribution of 20 measured channel cross-sections. The average shape size is $b=\overline{{\rm{BD}}}=112\pm 11$ nm, $c=\overline{{\rm{EC}}}=203\pm 16$ nm and $a=\overline{{\rm{E}}{\rm{B}}}=\overline{{\rm{B}}{\rm{C}}}=\overline{{\rm{C}}{\rm{D}}}=\overline{{\rm{D}}{\rm{E}}}=115\pm 22$ nm. The angles are $2\kappa =117\pm {8}^{\circ }$ and $2\lambda =63\pm {4}^{\circ }$. From SEM micrographs the number of pores per area and the mean cross-section area of the pores are determined. The distribution of the cross-section area is shown in Fig. 1c, its mean value is $A=\mathrm{(10.9}\pm \mathrm{1.2)}\times {10}^{3}$ nm^2^. The resulting porosity was determined to be $\mathrm{(3.4}\pm \mathrm{0.2)}\times {10}^{-4}$. The rhombi are all oriented along the same direction. The rhombius-shape is due to the oxygen planes of the crystal which have the slowest etching rate and build up the wall of the pores. The walls are thus parallel to the {110}-planes of the muscovite crystal^7^. To avoid charging effects the surface of the sheet was sputter coated with a 8 nm thick gold layer.

All ion beam transmission measurements were performed at the particle accelerator laboratory LIPSION at Universität Leipzig^15^. The mica sheet was mounted on a goniometer that enables an eucentric rotation in five directions^16^ with a resolution of less than 1 μm for *x*, *y*, *z* and 0.015° in *ϑ* and 0.005° in *φ*. These angles get projected on the sample in the following way: $\alpha =\vartheta \,\sin \,\phi$, where *α* is the angle between the ion beam and the pore direction, *ϑ* is a rotation perpendicular to the ion beam and *φ* is a rotation around the ion beam. The thickness of the mica sheet was determined by means of scanning transmission microscopy (STIM) using H^+^ ions at a kinetic energy of 2.25 MeV. The beam was focused to below 1 μm and completely penetrated the sheet. The corresponding energy loss of the transmitted ions was compared to a SRIM-2013^14^ calculation yielding a sheet thickness of $t=\mathrm{(7.0}\pm \mathrm{0.5)}$ μm. SRIM calculations do not take into account channeling effects, but this should not influence the result because hydrogen at such a high energy has a very small critical angle for crystal channeling of about 0.52° and due to a possible small misalignment and ion deflection from the gold layer on top of the sheet channeling is very unlikely. The STIM measurement across the entire sample also allowed us to confirm the mica sheet was intact and had no cracks from the preparation or mounting process.

**Figure 4:**
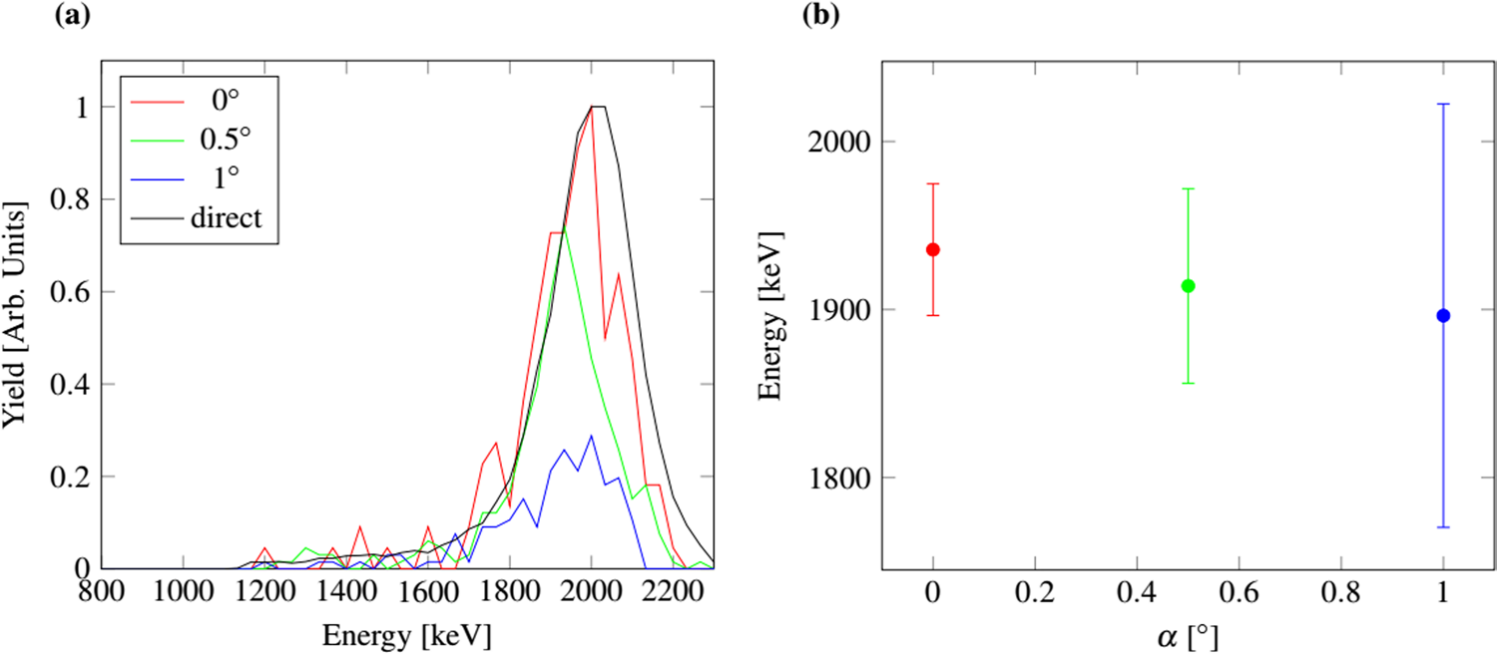
(**a**) Recorded number of N^2+^ ions with an initial energy of 2 MeV, as recorded with a surface barrier detector after transmission through the capillaries in the muscovite sheet under various angles between sheet and ion beam. The direct curve was measured without a sheet. The maximum of the direct peak and of the peak measured for 0° are normalized to one. The other two peaks are normalized to the 0° peak. The angle uncertainty is ${\rm{\Delta }}\phi =\pm {0.005}^{\circ }$. (**b**) Mean energy of the transmitted ions with corresponding measurement error.

To investigate the interaction between ions and capillaries at different kinetic energies, the experiments were performed with 800 keV N^+^, 2 MeV and 4 MeV N^2+^ ions. The beam flux was between 1.7 × 10^6^ and 0.1 × 10^6^ ions/cm^2^ s. In order to ensure a parallel beam, these measurements were performed without any focusing lens. The divergence angle of the ion beam is given by the distance and size of the beam defining apertures yielding 0.2 mrad. According to SRIM, the penetration depth of 4 MeV nitrogen ions in mica is 3 μm. For all beams the mica thickness was thus sufficiently large to completely stop the ions. A two axis goniometer was used to vary the angle of the sample surface with respect to the incoming ion beam. The transmitting ions and their energy were recorded by a surface barrier detector that was mounted behind the sheet. In a first step, the length axis of the pores was aligned parallel to the incident ion beam. Perfect alignment of the pores was assumed at the angle where maximum transmission is monitored. For the subsequent measurements, the angle *φ* was kept constant while the angle *ϑ* was varied. For every *ϑ* angle position, the irradiation time was the same and the number of ions passing the sheet was integrated. Figure 4 shows the recorded number of ions of 2 MeV N^2+^ after transmission through the capillaries as a function of their energy for various *ϑ* angles.

The radiation stability of the mica sheet was checked by inspecting the surface before and after the ion bombardment by SEM. The structure of the sheet was controlled with scanning transmission ion microscopy measurements using 2.25 MeV H^+^ ions. Several hours of ion irradiation with H^+^, N^+^ and N^2+^ ions of different energies showed no degradation and change on the muscovite. The datasets generated and analyzed during the current study are available from the corresponding author on reasonable request.

## References

[1] Lanyon YH (2007). Fabrication of nanopore array electrodes by focused ion beam milling. Anal. Chem..

[2] Toulemonde M, Bouffard S, Studer F (1994). Swift heavy ions in insulating and conducting oxides: tracks and physical properties. Nucl. Instrum. Methods Phys. Res. Sect. B.

[3] Pezzagna S (2011). Creation of colour centres in diamond by collimated ion-implantation through nano-channels in mica. Phys. Status Solidi A.

[4] Dolde F (2013). Room-temperature entanglement between single defect spins in diamond. Nat. Phys..

[5] Dolde F (2014). High-fidelity spin entanglement using optimal control. Nat. Commun..

[6] Toimil-Molares ME (2012). Characterization and properties of micro- and nanowires of controlled size, composition, and geometry fabricated by electrodeposition and ion-track technology. Beilstein J. Nanotechnol..

[7] Chien C (2002). Electrodeposited magnetic nanowires: arrays, field-induced assembly, and surface functionalization. J. Magn. Magn. Mater..

[8] Zhang H-Q (2012). Tailoring of keV-ion beams by image charge when transmitting through rhombic and rectangular shaped nanocapillaries. Phys. Rev. Lett..

[9] Wang G (2015). Transmission of hundred-keV protons through insulating nanocapillaries: Charge-patchassisted specular reflections. Sci. Rep..

[10] Gemmell DS (1974). Channeling and related effects in the motion of charged particles through crystals. Rev. Mod. Phys..

[11] Radoslovich E (1960). The structure of muscovite, kal2 (si3al) o10 (OH) 2. Acta Crystallogr..

[12] Navrotsky, A. *Mineral Physics and Crystallography - A Handbook of Physical Constants* (American Geophysical Union, Washington DC, 1995).

[13] Oura, K. *et al.**Surface Science* (Springer, Berlin, 2003).

[14] Ziegler, J. F., Biersack, J. P. & Ziegler, M. D. Srim-2013 (stopping and range of ions in matter), www.srim.org.

[15] Vogt J (2000). Solid state analysis with the new leipzig high-energy ion nanoprobe. Microchim. Acta.

[16] Nilsson C, Petriconi S, Reinert T, Butz T (2007). The new target chamber at lipsion: The new translation stage and goniometer and the new irradiation platform for single cell experiments. Nucl. Instrum. Methods Phys. Res., Sect. B.

